# Hypertension in Patients With Peripheral Artery Disease: An Updated Literature Review

**DOI:** 10.7759/cureus.62246

**Published:** 2024-06-12

**Authors:** Andrew T Abraham, Sanaullah Mojaddedi, Isaac H Loseke, Christopher Bray

**Affiliations:** 1 Graduate Medical Education/North Florida Regional Medical Center, University of Central Florida College of Medicine, Gainesville, USA

**Keywords:** maximal walking distance, claudication time, ankle-brachial index, hypertension, peripheral artery disease

## Abstract

Peripheral artery disease (PAD), a condition where there is reduced blood flow due to narrowing or blockage of the arteries of the peripheral vasculature, is an epidemic that currently affects eight million people in the United States alone and is a major risk equivalent to having active coronary artery disease (CAD). However, it is commonly underdiagnosed in the general population. Hypertension is a common cardiovascular condition characterized by elevated blood pressure levels. There are several mitigating risk factors that can reduce the risk of complications of PAD, with hypertension playing a major role. This literature review aims to explore the relationship between hypertension and PAD, including their shared risk factors, pathophysiological mechanisms, and management strategies. In addition, we will analyze how this impacts major cardiovascular outcomes, such as critical limb ischemia, vascular amputation, myocardial infarction (MI), ischemic stroke, and cardiovascular-related death by examining relevant studies, current guidelines, and evidence. This literature review is intended to guide practitioners on ideal blood pressure parameters and evidence-based anti-hypertensives that provide overall cardiovascular benefit in both the primary care and hospital-based setting. By understanding the association between hypertension and PAD and the underlying pathophysiological mechanisms, healthcare professionals can improve diagnosis, treatment, and management strategies for affected individuals.

## Introduction and background

Hypertension and peripheral artery disease (PAD) are two prevalent cardiovascular conditions that often coexist and significantly impact patient health outcomes. Hypertension, commonly known as high blood pressure, is a chronic medical condition characterized by elevated arterial blood pressure, while PAD refers to a condition where there is a narrowing or blockage of the peripheral arteries that supply blood to the legs, arms, stomach, or head. Patients who have PAD often have comorbid cardiovascular disease. Data suggest that there is a correlation of PAD severity and death from myocardial infarction (MI) and cerebrovascular accident (CVA) 1,2].

## Review

Shared risk factors

Numerous risk factors contribute to the development of both hypertension and PAD, with several commonalities between the two conditions. The most notable shared risk factor is advancing age, as both conditions are more prevalent in older individuals. Diabetes mellitus and smoking consign the highest risk of morbidity and mortality [3]. Other common risk factors include obesity, dyslipidemia, family history of vascular disease, and a sedentary lifestyle. These risk factors have been consistently identified in studies examining the association between hypertension and PAD, suggesting a shared etiology. Interestingly, women seem to have a higher prevalence of PAD than men. Moreover, PAD is also prevalent in diseases, such as obstructive sleep apnea, atrial fibrillation, congestive heart failure, and chronic kidney disease (CKD), which are all conditions with an accompanying relationship to hypertension [1,2].

Pathophysiological mechanisms

**Figure 1:**
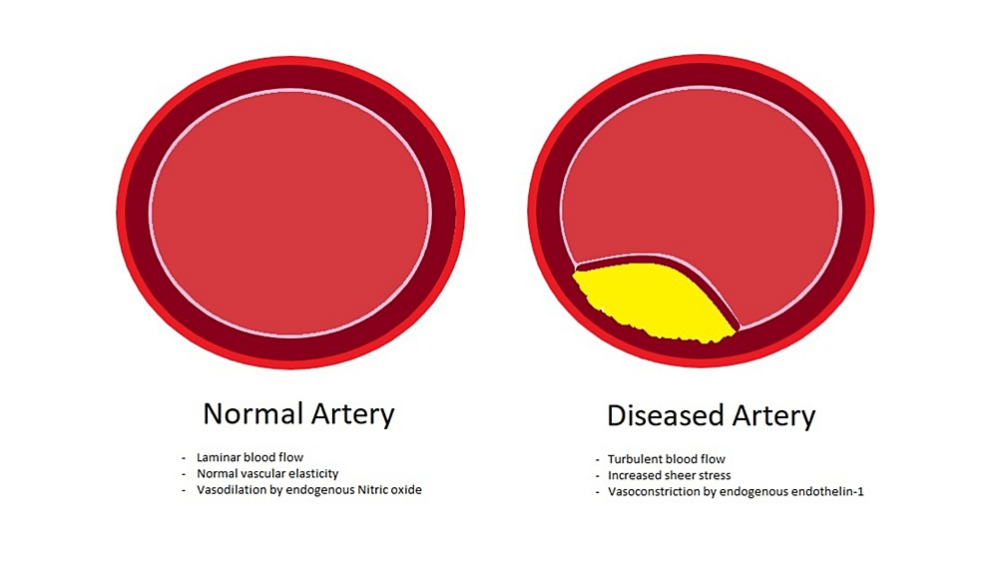
Image demonstrating 2D views of a normal blood vessel (left) and blood vessel with unruptured atheroma (right)

**Figure 2:**
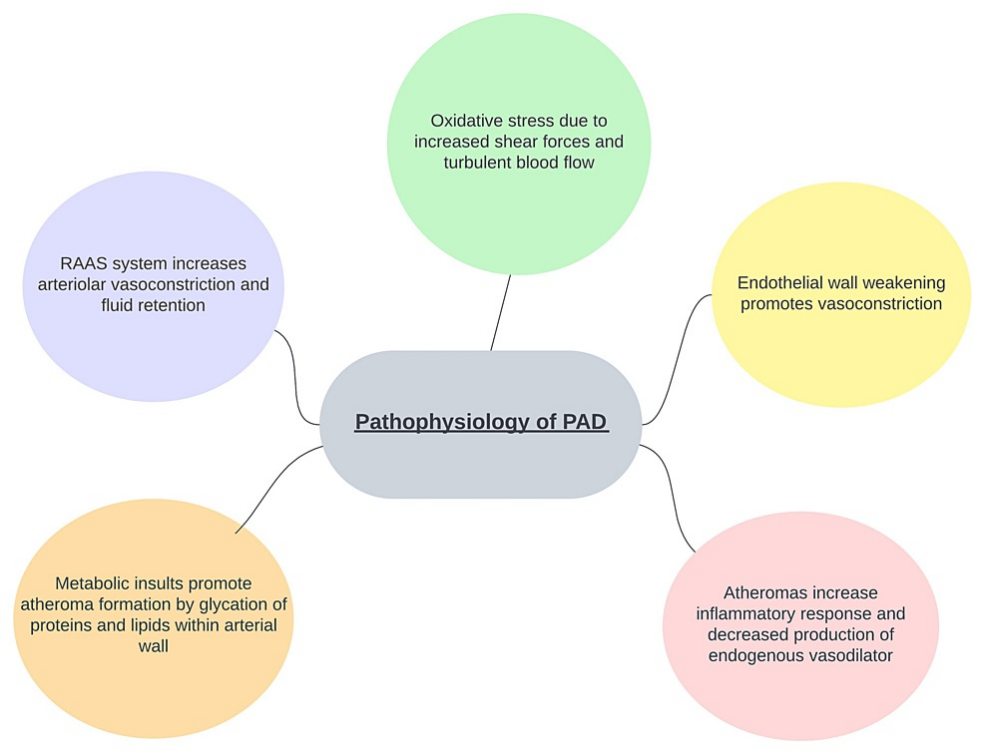
Graphic illustration of the pathophysiology of peripheral artery disease PAD, peripheral arterial disease; RAAS, renin-angiotensin-aldosterone system Citation: [1-3,5]

PAD has multiple underlying pathophysiology mechanisms, and hypertension and PAD share several. Endothelial dysfunction is a key contributor to both conditions. Elevated blood pressure and sheer stress on the arterial walls result in endothelial injury and impairment of vasodilation, leading to reduced blood flow. This reduced blood flow can exacerbate atherosclerotic processes in the peripheral arteries, contributing to the development and progression of PAD. Inflammatory processes, oxidative stress, and the renin-angiotensin-aldosterone system also play significant roles in the pathophysiology of both hypertension and PAD [1,2]. In addition, metabolic conditions, such as diabetes mellitus, cause glycation of proteins and lipids, which accelerate the formation of atheromas (Figure 1). These atheromas weaken the endothelium and lead to downstream reduction of nitric oxide, a vasodilator, and increased production of endothelin-1, a vasoconstrictor, and this only exacerbates these processes [4]. Understanding the pathophysiologic progression of this disease provides the roadmap to therapeutic interventions (Figure 2).

Classification and clinical presentation

The clinical presentation of PAD has been tiered into its four clinical presentations: asymptomatic, chronic symptomatic, chronic-limb-threatening ischemia, and acute limb ischemia (ALI). Asymptomatic PAD is classified as those who have no exertional claudication during an objective walking test. Chronic symptomatic PAD comprises claudication symptoms that worsen with exertion and typically resolve at rest and is the most commonly encountered presentation. Chronic limb-threatening ischemia (CLTI) presents with ischemic rest pain present for <2 weeks in addition to skin breakdown, such as wounds and gangrene. The Fontaine stages and Rutherford classification systems are used to stratify severity of CTLI and is used frequently by vascular specialists. The Wound, Ischemia, and Foot Infection (WIfI) classification is used to estimate the risk of lower extremity amputation in those with diabetic foot disease, ischemia, and infection and can be used in conjunction with the Fontaine and Rutherford classification systems for the assessment of vascular intervention. ALI is often recalled by the “6 P’s”: pain, pallor, pulselessness, poikilothermia (coolness), paresthesia, and possible paralysis occurring <2 weeks. The Rutherford classification system is also used in the stratification of ALI. Understanding the nomenclature helps in distinguishing the prognosis in a variety of PAD presentations [5].

Association and impact

Epidemiological studies have consistently demonstrated a strong association between hypertension and PAD. Patients with hypertension have a higher prevalence of PAD, and the presence of both conditions leads to worse outcomes and an increased risk of cardiovascular events [2,6]. Hypertension exacerbates the progression of atherosclerosis in the peripheral arteries, leading to decreased peripheral perfusion, increased risk of critical limb ischemia, and impaired wound healing. Furthermore, hypertension and PAD often coexist with other cardiovascular conditions, such as coronary artery disease and stroke, further compounding their impact on patient health [1]. To see this application, look no further than the DAPHNE study, a prime example of how hypertension plays a pivotal role in peripheral atherosclerosis. Doxazosin is an alpha-blocker that lowers blood pressure through alpha-adrenergic blockade of peripheral vessels that also has the benefit of lowering plasma lipids. This agent was compared with hydrochlorothiazide, a diuretic that affects blood pressure but increases resistance to insulin. Investigators looked at changes in arterial intimal wall thickness and cholesterol changes. At the conclusion of the study, it was found that both agents demonstrated significant improvement in arterial wall thickness, despite the differences in plasma lipid effects [7]. While blood pressure goals are generally uncertain, the significant impact of blood pressure extremes seem to have an overall negative impact on peripheral artery disease. However, it was found that patients who had hypertension were ubiquitous with having PAD that was asymptomatic [8]. Therefore, the link with blood pressure and PAD is no doubt a strong one. Given the significant association of PAD to MI and stroke, the European Society of Cardiology considers PAD patients to be at a significant overall risk for cardiovascular complications [9].

Racial disparities

Hypertension is known to lead to a multitude of long-term complications, with one of those being PAD. Treatment throughout the world is variable and dependent on cultural, social, and financial factors. The prevalence of PAD is greater in high-income countries, but from 2000 to 2015, the prevalence of PAD has increased by approximately 58% in low-income and middle-income countries [10]. In low-income and middle-income countries, PAD seemed to be most prevalent in higher in women and in countries within Southeast Asia and the western Pacific. While the prevalence of intermittent claudication is higher in men, women and non-Caucasian ethnicities seemed to report intermittent claudication symptoms less frequently than men. While severe ABI values generally correlate with severity of PAD, rates of critical limb ischemia leading to amputation were found to be variable throughout the world. Questionnaires could be a reasonable and cost-effective measure to assess for the presence of PAD symptoms. The San Diego Claudication Questionnaire and the Edinburgh Claudication Questionnaire has exceptional specificity. If the results of these questionnaires have a positive result, further workup can be done. While cigarette smoking and diabetes remain the most significant risk factors increasing risk of PAD, studies have shown that the risk collectively increases with multiple risk factors present. With hypertension becoming more prevalent in low-income and middle-income countries, the impact of PAD will subsequently become more profound [11].

A recent meta-analysis found that while the prevalence of PAD increased with age, younger people in lower-income and middle-income countries had higher prevalence of PAD than younger people in high-income countries [12]. Women tend not to exhibit typical PAD symptoms or may not even have any claudication symptoms at all. Therefore, women may not even be aware that they have PAD. In low-income countries, more women have PAD than men, despite smoking being more prevalent among men. In addition, the prevalence of PAD seems to be higher in nonwhite women versus white women [13]. Among the Black population throughout the world, there is sparse data on the epidemiology of PAD compared between geographic regions. Black Americans had significantly more PAD as than any other racial groups, particularly after age 50 years old. Despite accounting for primary risk factors of PAD, Black Americans still remained at substantial risk of having PAD. This is likely due to the higher prevalence of risk factors, in particular rates of hypertension being highest in Black American. However, rates of treatment for hypertension are the lowest in Black Americans. In addition, Black Americans have higher prevalence of asymptomatic PAD and tend to have more severe disease and worse outcomes. The “Hispanic paradox” is the singularity of hispanic Americans having lower prevalence of PAD than non-Hispanic White Americans, but having higher prevalence of diabetes, which is one of the primary risk factors of PAD. Among Hispanic ethnicities, Puerto Ricans had the highest prevalence of PAD.

A study of over 96,000 Native Americans found that PAD rates were higher than non-Hispanic White’s [14]. In a study of Chinese people with hypertension, it was found that the prevalence of PAD in those with hypertension was higher than those without hypertension. However, it was found that diabetes and CAD was not associated with PAD among this population [15]. Those who had hypertension during pregnancy had an increased risk of developing PAD decades after pregnancy, despite having ever smoked [16]. While PAD affects a broad population, Black Americans are significantly impacted by this and consequently have worse outcomes.

Diagnostic evaluation

Although getting a thorough history and physical is tantamount, oftentimes, the patients may not have symptoms. Those with moderate to severe burden of disease may present with intermittent claudication, skin changes, and weak or absent pulses. The ankle-brachial index (ABI) and Doppler pulses should be the next step in the evaluation. A value of ≤0.9 is diagnostic for PAD, 1.00-1.4 is normal, and values ≥1.40 are nondiagnostic as these reflect over-calcified vessels that are noncompressible. In addition, an elevated C-reactive protein is strongly linked to an increased risk for PAD [6]. When ABI values are normal or nondiagnostic and the suspicion for PAD still remains elevated, there are several other noninvasive and relatively cheap options that can be explored. Pulse volume recorder (PVR) uses Doppler ultrasound along with the blood pressure cuff simultaneously. Furthermore, patients with non-compressible values on ABI may show positive findings on PVR. Duplex ultrasound uses sound waves to get a live dynamic view of vascular flow. Transcutaneous oxygen pressure monitoring evaluates the delivery of oxygen to tissues by placing skin probes at selected spots and is a reliable measure of limb ischemia and can predict adequate wound healing after amputation. Computerized tomography (CT) and magnetic resonance (MR) angiography offer the most detailed view of limb ischemia, as well as severity of lesions, precise locations, and collateral circulation. It is the imaging modality of choice to evaluate PAD prior to intervention. The diagnostic gold standard is digital subtraction angiography (DSA), but it is rarely used due to potential complications and because CT angiography is not inferior to DSA in the preoperative setting [2].

Prognostic vitals and lab values

Hypertension is a known risk factor for PAD. However, can blood pressure readings prior to revascularization be prognostically important? A retrospective cohort study looked at patients undergoing revascularization for critical limb ischemia and found that admission systolic blood pressures were correlated with mortality. In particular, SBPs less than 135 were associated with increased mortality [17]. Another study looking at prognostic vitals found that resting heart rate correlated to mortality in PAD and heart rates >75 bpm increased the risk of all-cause mortality [18]. C-reactive protein (CRP) is a biomarker of inflammation and is commonly elevated in cardiovascular diseases. In relation to PAD, the CRP-to-albumin ratio has been proposed as a prognostic marker in PAD. It was found that these levels were strongly associated with all-cause mortality in PAD and amputation after endovascular intervention [19]. In addition, in patients who underwent endovascular intervention, the CRP-to-albumin ratio was found to be strongly associated with complex lesions at multiple levels of the lower extremity, increased incidence of major adverse cardiac events, and major adverse limb events after endovascular intervention [20]. Another study looked at N-terminal pro-b-type natriuretic peptide (NT-proBNP), high-sensitivity cardiac troponin T, and ischemia-modified albumin in male patients with PAD with Fontaine stage 2. It was found that ischemia-modified albumin was a strong predictor of major adverse cardiac events in this population and could be an independent predictor of morbidity [21]. Urinary cystatin C is a marker classically associated with kidney function. However, the elevated urine cystatin C:urine creatinine ratio is associated with PAD and is a predictive of major adverse limb events [22]. During angiography, myeloperoxidase levels were assessed in patients with PAD and CAD. Higher myeloperoxidase levels were associated with higher rates of limb ischemia leading to revascularization [23]. A systematic review looked at interleukins (ILs) as a diagnostic marker in PAD. Based on the data, IL-6 and IL-8 are the most associated with PAD prognosis as these patients tend to have higher plasma levels of these pro-inflammatory molecules [24]. These prognostic tools can be valuable tools in the outpatient and inpatient clinical setting.

Current evidence on blood pressure goals

Hypertension is prevalent in over one-third of all patients with PAD. However, many experts have argued on what the ideal blood pressure targets should be. Having a higher blood pressure may allow for adequate distal perfusion, but this compromises long-term cardiovascular outcomes, whereas a lower blood pressure goal can mitigate this risk but potentially jeopardizes perfusion to the diseased area. Based on prior studies, intensive blood pressure goals have been shown to worsen limb symptoms in the PAD population [25]. In the ATTEST Study, recruited patients were classified into blood pressure ranges between normotensive, isolated systolic hypertensive, and concomitant systolic and diastolic hypertension, with normotensive being an SBP <140 mm Hg and DCP <90 mmHg. It was found that isolated systolic hypertension is one of the central issues that negatively impacts secondary cardiovascular prevention, particularly those with PAD [26]. The HOPE (Heart Outcomes Prevention Evaluation) study found that antihypertensives can reduce cardiovascular morbidity and mortality in patients with PAD [27]. The PORTRAIT registry, an observational study on blood pressure management in patients with PAD, looked at patients on different antihypertensive medications and found sub-optimal blood pressure management due to the heterogeneity in “normal” values in PAD [28]. However, the data comparing higher BP ranges against lower BP ranges have been incongruous.

The SPRINT (Systolic Blood pressure Intervention Trial) trial demonstrated that tightly regulated blood pressure control with SBP <120 had less risk for cardiovascular events in PAD versus an SBP range of 135-139 [29]. However, post-hoc analysis of the INVEST (International Verapamil-SR/Trandolapril Study) trial found that an SBP range of 135-145 had lower rates of all-cause death, non-fatal MI, and stroke in patients with CAD and PAD. The post hoc-analysis of the ALLHAT (Antihypertensive and Lipid-Lowering Treatment to Prevent Heart Attack Trial) trial specifically looked at the impact of blood pressure and pulse pressure on lower-extremity PAD events. The results demonstrated higher PAD events with SBP <120 and DBP <70 [27,30]. The EUCLID Trial in 2020 included over 13,000 patients with symptomatic PAD and approximately 78% had hypertension. It was found that when systolic blood pressure was increased by intervals of 10 mmHg from 125 mmHg, it was associated with increased risk of major adverse cardiovascular events and major adverse limb events and increased need for lower extremity revascularization. Furthermore, systolic blood pressure drops by intervals of 10 mmHg from 125 mmHg were associated with major adverse cardiovascular events but not major adverse limb events or need for revascularization. The authors concluded that adverse cardiovascular events are associated with an out-of-target range for blood pressures. Interestingly, a history of hypertension and event rates were not associated in this study [31].

Several retrospective studies have looked at the relationship between blood pressure following revascularization. Preoperative pulse pressure of ≥80 mmHg was associated with decreased amputation free survival at six months and one year. Moreover, another study looking at below-knee interventions in critical limb ischemia found an increased amputation rate in patients with controlled blood pressures, which were considered to be ≤140/90. Despite these findings, continuing an antihypertensive therapy to target is recommended in the majority of cases [8]. Moreover, current guidelines recommend treating patients with PAD to a target of <130/80 mm Hg while keeping in mind that overtreatment may lead to worse outcomes [5]. While we recognize the risk of hypertension in PAD, low DBP is also associated with higher cardiovascular complications.

Management strategies

The optimal management of hypertension and PAD requires a comprehensive approach that targets shared risk factors and addresses the specific needs of each condition. Lifestyle modifications, including regular exercise, weight loss, smoking cessation, and a heart-healthy diet, are cornerstone interventions for both conditions. Hemoglobin A1c levels less than 8 correlate favorably to improved outcomes [32]. Pharmacological management typically involves a multimodal approach. Antiplatelet therapy, including aspirin or P2Y12 receptor inhibitor, is commonly prescribed to reduce cardiovascular events in patients with both hypertension and PAD. Cilostazol is the only approved pharmacological treatment for intermittent claudication. Statins have been found to reduce the risk of worsening PAD (2) and is a class 1A recommendation [33]. In addition, other lipid-lowering therapies, such as ezetimibe and PCSK9 inhibitor, show promise in further reducing the lipid load [4]. Rivaroxaban, a factor Xa inhibitor, in conjunction with aspirin was found to reduce major cardiovascular outcomes, such as MI and stroke, as evidenced from the COMPASS trial [4] and significantly reduced ALI after revascularization, as evidenced by the VOYAGER-PAD trial [34]. In severe cases, endovascular intervention or surgical revascularization may be necessary to restore blood flow in the affected peripheral arteries. Given this, hypertension is a significant independent variable that has consequential outcomes in PAD, and treatment for such is a recommendation per the 2024 ACC guidelines [5]. Table 1 goes into further details of each antihypertensive medication and the evidence of each agent in this population.

**Table 1:** Anti-hypertensive agents in peripheral artery disease (PAD)

Anti-hypertensive agents	
Angiotensin-converting enzyme (ACE) inhibitors/angiotensin receptor blockers (ARB)	Angiotensin-converting enzyme (ACE) inhibitors and angiotensin receptor blockers (ARB) share a common pathway. ARB’s displace angiotensin-2 from its receptor sites, limiting its systemic effects, such as vasoconstriction, sodium reabsorption and water retention, sympathetic activation, and cardiovascular remodeling via aldosterone. ACE inhibitors block the enzyme that converts angiotensin-1 to angiotensin-2, effectively halting these downstream results. These medications, through their shared mechanisms, contribute to progressive vasodilation and natriuresis and improve hypertension [35]. The HOPE study found that patients taking ramipril had significantly fewer cardiovascular adverse outcomes [36]. The ONTARGET (Ongoing Telmisartan Alone and in Combination With Ramipril Global End Point Trial) trial found no significant difference between telmisartan and rampiril in regard to primary cardiovascular events, such as MI, stroke, or hospitalization for heart failure [4]. Another randomized controlled trial of 36 patients on telmisartan with mild to moderate hypertension showed remarkably improved walking distance and ABI values [37]. In concomitant renal artery disease, renin-angiotensin-aldosterone antagonists, CCBs, BBs, and diuretics are effective agents at controlling blood pressure, but ACE inhibitors and ARBs have shown a mortality benefit in this population [38]. This evidence additionally points to a potential patho-pharmacologic mechanism for vascular preservation. ACE inhibitors and ARBs are considered first-line options for patients with hypertension and PAD per the 2024 ACC/AHA guidelines [5].
Calcium-channel blockers (CCBs)	Calcium-channel blockers (CCBs) inhibit calcium from entering the cells of vascular smooth muscle of the cardiac muscle. The dihydropyridines primarily work on the vascular smooth muscle, leading to the vasodilation and reduction of blood pressure. The non-dihydropyridines mainly act on the cardiac myocytes by reducing chronotropy and subsequently reducing the cardiac output and blood pressure. In addition, CCBs have natriuretic properties through the vasodilation of renal afferent arterials in the glomerulus and increasing glomerular filtration. Non-dihydropyridines are selective in reducing albuminuria [39]. A meta-analysis of trials pooled after 1990 attempted to assess the effect of CCBs in patients with HTN for the primary prevention of PAD. Seven trials met the inclusion criteria and patients within the control group received either ACE inhibitors, diuretics, beta-blockers, or placebo. They found that those who received CCBs were less at risk for developing PAD versus the control group. In addition, a subset analysis found that dihydropyridine CCBs were associated with 26% risk reduction in PAD (95% CI, p < 0.001) [40]. They concluded that CCBs, particularly dihydropyridines, seemed to be efficacious for the primary prevention of PAD in patients with HTN. Another meta-analysis evaluated CCB and the development of PAD and found a 30% reduction in the odds in those taking CCB. In addition, those with established PAD had fewer coronary artery adverse outcomes. This can be explained by the possible mechanism of CCBs with the reduction of atherosclerosis within the vascular beds, which have been previously demonstrated in animal studies [41]. In carotid atherosclerosis, CCBs and ACE-Is are recommended, but data favor CCBs due to the slowing of carotid intima-media thickening [38]. Several experimental studies have demonstrated this suppression of vascular atherosclerosis [42,43,44]. Given the pleiotropic effects in atherosclerosis reduction within coronary and peripheral vasculature and its antihypertensive properties, CCBs appear to be a practical and safe option in patients with HTN and PAD.
Chlorthalidone	Chlorthalidone is a thiazide diuretic that was commonly looked over in favor of the more recognized hydrochlorothiazide (HCTZ). However, recent studies have shown the efficacy of chlorthalidone on blood pressure and its potential cardioprotective and renoprotective effects. A meta-analysis compared chlorthalidone and HCTZ and found that chlorthalidone was superior due to its prolonged effect on blood pressure. This is likely because of its longer half-life and larger volume of distribution [45]. Examining PAD events within the ALLHAT trial, the CCB Amlodipine and the ACE-inhibitor Lisinopril was not superior to chlorthalidone. Moreover, between the three groups, there was no significant difference for nonfatal myocardial infarction, coronary revascularization, strokes, heart failure, or other mortality outcomes [46]. As mentioned above, many patients exhibit CKD and PAD concurrently. The Chlorthalidone for Hypertension in Chronic Kidney Disease (CLICK) trial examined patients in stage 4 CKD and poor controlled hypertension and found significant improvement of SBP over 12 weeks compared with placebo. Furthermore, reduced albuminuria was observed, pointing to its potential renoprotective qualities [47]. In addition to its longer half-life, chlorthalidone also promotes natriuresis, which has potential in mitigating hypertension in PAD. Therefore, chlorthalidone remains a reasonable and effective option.
Beta-blockers	The correlation is strong between PAD and CAD. As such, it is common to see patients with diagnosed CAD on long-standing beta-blocker therapy. However, the therapeutic effect of beta-blockers comes from reducing blood flow and pressure and theoretically worsening PAD. However, a meta-analysis demonstrated that claudication symptoms did not worsen while on beta-blockers. A prospective observational study found that patients with myocardial infarction and intermittent claudication not on beta-blocker therapy had worse outcomes than those who were on it [48]. A meta-analysis conducted in 2013 evaluated selective (β1) and non‐selective (β1 and β2) beta-blockers for PAD. The agents analyzed were atenolol, propranolol, pindolol, and metoprolol, with the primary outcome being maximal walking distance and claudication time and distance. The secondary outcomes included calf blood flow and vascular resistance. The authors found that these agents did not worsen the maximal walking distance or time to claudication nor did it worsen calf blood flow or calf vascular resistance [49]. There were several limitations noted by the study authors, but this provides a launchpad for further investigational research. As with thiazides, beta blockers, particularly propranolol, are associated with decreases in insulin secretion and sensitivity, which can worsen metabolic derangements that could contribute to PAD [50]. Nevertheless, beta-blockers should be considered in patients with PAD with prior myocardial infarction for cardioprotective effects, as it was shown in multiple studies that cardiovascular mortality and coronary events without having an impact on amputation rates or worsening or claudication symptoms [38]. Although more studies are needed, beta-blockers and alpha-blockers that preferentially improve peripheral blood flow may show benefit in the future.

Emerging therapies

While pharmacotherapy targeting hypertension is tantamount in controlling the progression of PAD, there are other therapies that may serve in tandem to improve symptoms. Several studies have shown the benefits of inorganic nitrates with intermittent claudication. These can be found in beetroot juice and appear to reduce systolic blood pressure by preventing oxygen free-radicals from inhibiting endogenous nitric oxide. Mitochondria-targeted antioxidants, which can be found in dark chocolate, have also shown benefit with walking distance in PAD. The antidiabetic drug metformin has been shown to improve lower extremity blood flow [4]. However, endothelin-receptor antagonist and the anti-anginal agent ranolazine have demonstrated mixed reviews and can be considered with additional comorbidities [1]. In addition, there have been several emerging therapies that are gaining recognition in the field of PAD. Autologous stem cell therapy has been shown to improve PAD-associated ulcer healing, but more in-depth studies are needed. While, gene therapy targeting angiogenesis may point to a radical new remedy, the data were not entirely favorable [4]. In particular, data from studies looking at hepatic growth factor have been encouraging in ameliorating ulcer size [1]. Endovascular interventions directly target the diseased area with transluminal angioplasty. The balloon systems available can not only expand the arterial area, but newer devices have the ability to induce vasodilation with cryotherapy, inhibit medial hyperplasia with drug-coated balloons, or rupture calcified plaques with soundwaves [3]. Until most recently, chronic limb-threatening ischemia required emergent amputation. However, the PROMISE 2 trial provided a novel transcatheter technique that involves creating an arteriovenous fistula between the diseased artery and nearby deep veins. The oxygen-rich blood is then diverted to the distal vasculature by way of the venous system. This has been found to effectively improve limb salvage [51].

Endovascular intervention versus conventional medical therapy

Endovascular intervention has revolutionized the management of PAD. However, how does it compare against conventional medical therapy? It was found that endovascular revascularization in patients with intermittent claudication put patients at higher risk for repeat intervention compared with conservative management. Despite this, several meta-analysis’ have confirmed that the combination of a supervised exercise program in conjunction with endovascular intervention targeting revascularization led to the most benefit compared to each individually alone in one- to two-year follow-up and reduces the risk of subsequent revascularizations [4]. It is important to factor cost against time-to-benefit when it comes to deciding on treatment. It stands to reason that management with a supervised exercise program, lifestyle modifications and aggressive risk factor mitigation with established strict blood pressure goals, statin initiation, and dynamic metabolic control should be the initial therapy of choice if feasible, as this will favorably reduce major cardiovascular events, such as critical limb ischemia, vascular amputation, MI, ischemic stroke, and cardiovascular-related death. However, in those with chronic limb-threatening ischemia who are not responsive to maximally tolerated medical therapy and supervised exercise therapy, revascularization should be utilized to prevent limb loss and increase quality and functionality of life [5].

## Conclusions

Hypertension and PAD frequently coexist and synergistically contribute to adverse cardiovascular outcomes. By conducting an updated literature review on hypertension and PAD, this study aims to provide healthcare professionals with valuable insights into the relationship between these two conditions. Lifestyle modifications and pharmacological interventions targeting hypertension and PAD are essential to reduce the risk of cardiovascular events and improve patient outcomes. Ultimately, patients with PAD should be prescribed medical therapies to reduced major adverse cardiovascular events and major adverse limb events, which include a single-antiplatelet agent with a possible antithrombotic agent, lipid-lowering therapy, anti-hypertensive therapy, and lifestyle modifications. The recommendations from the 2024 Guidelines state a systolic blood pressure target of <130 and a diastolic blood pressure target of <80. While no single antihypertensive medication appears to be more effective at treating hypertension in patients with PAD, ACE inhibitors and ARBs should be considered first line as these confer the most cardiovascular benefits based on the current evidence. A better understanding of this association can aid in the early detection, accurate diagnosis, and effective management of both hypertension and PAD, ultimately improving patient outcomes and reducing the burden of cardiovascular disease. Further research is warranted to explore novel therapeutic strategies and optimize the management of these interconnected conditions.
